# Down-regulation of miR-146b-5p by long noncoding RNA MALAT1 in hepatocellular carcinoma promotes cancer growth and metastasis

**DOI:** 10.18632/oncotarget.15640

**Published:** 2017-02-23

**Authors:** Chao Li, Runchen Miao, Sushun Liu, Yong Wan, Simin Zhang, Yan Deng, Jianbin Bi, Kai Qu, Jingyao Zhang, Chang Liu

**Affiliations:** ^1^ Department of Hepatobiliary Surgery, The First Affiliated Hospital of Xi’an Jiaotong University, Xi’an 710061, Shaanxi Province, China; ^2^ Department of Geriatric Surgery, The First Affiliated Hospital of Xi’an Jiaotong University, Xi’an 710061, Shaanxi Province, China

**Keywords:** microRNA-146b-5p, hepatocellular carcinoma, TNF receptor associated factor 6, growth, metastasis

## Abstract

MicroRNAs play an important role in liver cancer genesis and progression. In this study, we identified down-regulation of miR-146b-5p associated with tumor growth, metastasis and poor survival in hepatocellular carcinoma (HCC) patients. miR-146b-5p could suppress proliferation, migration, and invasion and induced apoptosis *in vitro* and *in vivo*. Remarkably, TNF receptor associated factor 6 (TRAF6) was confirmed as a direct target of miR-146b-5p in HCC and miR-146b-5p exerted the tumor suppression roles through inhibiting the phosphorylation of Akt mediated by TRAF6. Furthermore, we identified long non-coding RNA MALAT1 as a molecular sponge of miR-146b-5p to down-regulate its expression in HCC. In general, our results indicate that miR-146b-5p inhibits tumor growth and metastasis of HCC by targeting TRAF6 mediated Akt phosphorylation.

## INTRODUCTION

Hepatocellular carcinoma (HCC) is one of the most common digestive cancers in China [1]. MicroRNAs represent as a class of endogenous short single-chain RNAs that regulate various cellular processes through binding to the 3′ untranslated region (3′-UTR) of target mRNAs [2]. Plenty of reports have well established their roles in metabolism [3], growth [4], senescence [5], angiogenesis [6] and metastasis [7].

Data in mounting numbers revealed that miR-146b-5p was involved in cancer genesis and progression [8]. However, miR-146b-5p is a friend or foe that depends on the cancer types. Up-regulation of miR-146b-5p was found in osteosarcoma tissues [9], and it promoted proliferation, migration and invasion through inhibiting ZNRF3 in osteosarcoma cells. Nevertheless, miR-146b-5p inhibited glioma cells’ migration and invasion through silencing MMP-16 expression [10]; it also attenuated stemness and radioresistance through restraining HuR/lincRNA-p21/β-catenin pathway [11]. In papillary thyroid carcinoma (PTC), the role of miR-146b-5p was more equivocal. An elevated expression of miR-146b-5p was observed in PTC tissues [12, 13]. Over-expression of miR-146b-5p enhanced growth and metastasis of PTC cells [14, 15]. However, recent study discovered that exosomes derived from TPC-1 cells were rich in miR-146b-5p, but these exosomes served as a negative regulator for cell proliferation to both TPC-1 and normal thyroid follicular cells [16]. Even the role of miR-146b-5p has been uncovered in many cancers, the expression and functions of miR-146b-5p in HCC are still unclear.

In this study, we disclosed that miR-146b-5p was down-regulated in HCC tissues and correlated with poor prognosis. *In vitro* and *in vivo* experiments showed that miR-146b-5p could suppress proliferation, migration, and invasion and induce apoptosis through inhibiting TRAF6/p-Akt signaling pathway.

## RESULTS

### Decreased expression of miR-146b-5p is associated with malignant clinical features and poor prognosis

**Figure 1:**
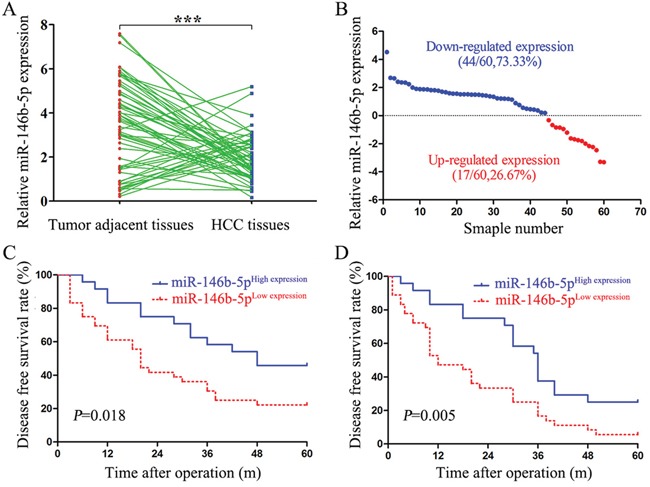
Low expression of miR-146b-5p in HCC tissues relates to poor prognosis **A**. The expression levels of miR-146b-5p in HCC tissues and matched tumor-adjacent tissues. ^**^*P<0.001. **B**. Down-regulation of miR-146b-5p was detected in 73.33% (44/60) of HCC tissues. **C** and **D**. Low expression of miR-146b-5p was significantly associated with the poorer 5-year overall survival (C, log-rank=5.606, *P*=0.018) and disease-free survival (D, log-rank=7.692, *P*=0.005) of HCC patients. Patients were divided into high and low expression subgroups according to the mean value of miR-146b-5p.

As shown in Figure 1A and 1B, 44 HCC tissues (73.33%) were detected down-regulated (log^NT/T^>0) miR-146b-5p expression, whereas only 17 HCC tissues had up-regulated (log^NT/T^<0) miR-146b-5p expression (*P*<0.001). We selected the mean level of miR-146b-5p (mean value=1.917) as a cut-off value to divide all 60 HCC patients into miR-146b-5p low expression group (n=35, <1.917) and high expression group (n=25, ≥1.917). As shown in Table 1, decreased expression of miR-146b-5p was associated with large tumor diameter (≥5cm, *P*=0.033), venous infiltration (*P*=0.046) and advanced TNM stage (stage III+IV, *P*=0.040). In this cohort, compared with the patients in high miR-146b-5p expression group, patients in low miR-146b-5p expression group had low 5-year overall survival (OS) rate (log-rank=5.606, *P*=0.018, Figure 1C) and disease-free survival (DFS) rate (log-rank=7.692, *P*=0.005, Figure 1D).

**Table 1:** Clinical correlation of miR-146b-5p expression in HCC (N=60)

Clinical Characteristics		No. of patients (N=60)	No. of patients (N=60)		χ^2^	*P*
			146b-5p^High^n=25	146b-5p^Low^ n=35		
Gender	Male	39	15	24	0.471	0.493
	Female	21	10	11		
Age (year)	<50	29	11	18	0.322	0.570
	≥50	31	14	17		
Tumor size (cm)	<5	24	14	10	4.571	0.033*
	≥5	36	11	25		
Liver cirrhosis	Absent	13	7	6	1.013	0.314
	Present	47	18	29		
Serum AFP level (ng/mL)	<400	20	11	9	2.194	0.139
	≥400	40	14	26		
Venous infiltration	Absent	42	21	21	4.000	0.046*
	Present	18	4	14		
Edmondson-Steiner grading	I+II	35	11	24	3.623	0.057
	III+IV	25	14	11		
TNM stage	I+II	39	20	19	4.239	0.040*
	III+IV	21	5	16		

*P<0.05.

Abbreviations: AFP, alpha-fetoprotein; TNM, tumor-node-metastasis.

### miR-146b-5p inhibits growth and metastasis in HCC cells

**Figure 2:**
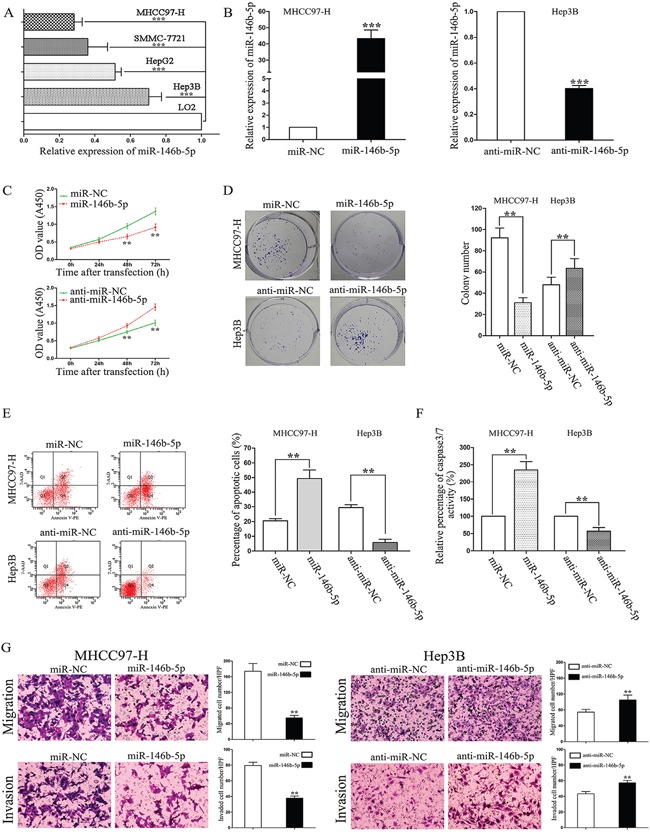
The effects of miR-146b-5p on proliferation, apoptosis, migration, and invasion of HCC cells **A**. Expression of miR-146b-5p in the human immortal liver cell line (LO2) and four HCC cell lines (MHCC97-H, SMMC-7721, Hep3B and HepG2). ^**^*P<0.001. **B**. Alteration of miR-146b-5p levels in HCC cells according their basal levels. miR-146b-5p expression levels were determined by qRT-PCR. ^**^*P<0.001. **C-G**. Alteration of miR-146b-5p expression levels markedly influenced cell viability (C), colony numbers (D), apoptotic cell percentages (E), caspase 3/7 enzyme activity (F), migration and invasion (G) in MHCC97-H and Hep3B cells. ^*^P<0.01. All experiments were performed at least in triplicate and the data in **A-G** are presented as the (mean ± SD).

We detected the expression levels of miR-146b-5p in four HCC cell lines (MHCC97-H, SMMC-7721, Hep3B and HepG2) and a human immortal liver cell line (LO2). As shown in Figure 2A, compared with the expression of miR-146b-5p in LO2, MHCC97-H and Hep3B had the lowest and highest expression levels of miR-146b-5p in these four cell lines, repectively (*P*<0.001, respectively). So we selected these two cell lines for further experiments. First, we stably over-expressed miR-146b-5p in MHCC97-H cells and knocked down miR-146b-5p expression in Hep3B cells (*P*<0.001, respectively, Figure 2B). Next, we concluded from CCK-8 assays and plate clone formation assays that miR-146b-5p over-expression suppressed cell viability and proliferation in MHCC97-H cells; on the contrary, inhibition of miR-146b-5p depressed cell viability and proliferation in Hep3B cells (*P*<0.01, respectively, Figure 2C and 2D). Additionally, determined by flow cytometry and caspase 3/7 activity assays, up-regulation of miR-146b-5p induced apoptosis in MHCC97-H cells and down-regulation of miR-146b-5p reduced apoptosis in Hep3B cells (*P*<0.01, respectively, Figure 2E and 2F). We further determined cell migration and invasion through transwell chambers. As shown in Figure 2G, up-regulation of miR-146b-5p significantly decreased the migration and invasion in MHCC97-H cells (*P*<0.01, respectively). On contrary, down-regulation of miR-146b-5p increased the migration and invasion in Hep3B cells (*P*<0.01, respectively).

### miR-146b-5p inhibits tumor growth and metastasis *in vivo*

**Figure 3:**
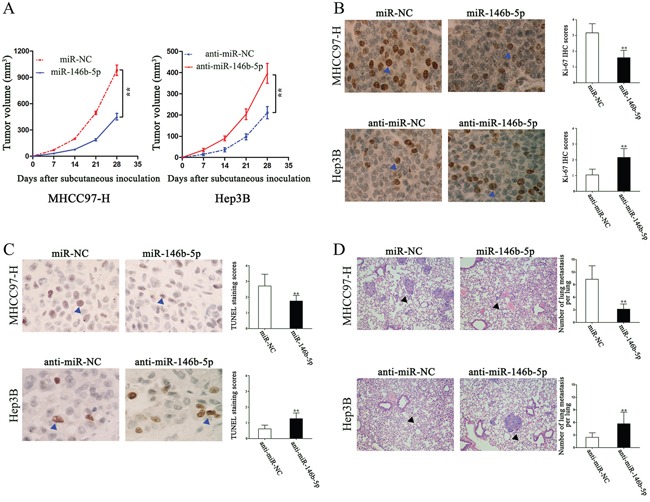
The *in vivo* effects of miR-146b-5p on tumor growth and metastasis of HCC **A**. As determined by tumor growth curves, miR-146b-5p over-expression markedly suppressed subcutaneous transplantation tumor growth; however, miR-146b-5p promoted tumor growth. n=six for every group. ^*^P<0.01. **B** and **C**. *In vivo*, miR-146b-5p inhibited MHCC97-H cells proliferation and induced apoptosis, whereas anti-miR-146b-5p promoted Hep3B cells proliferation and arrested apoptosis.^*^P<0.01. **D**. Lung metastatic clusters performing by tail vein injection are shown at 4 weeks. Over-expression of miR-146b-5p in MHCC97-H cells decreased their lung metastasis; miR-146b-5p knockdown increased the lung metastasis of Hep3B cells. ^*^P<0.01. Data in **A-D** are presented as the (mean ± SD).

In order to further confirm our *in vitro* results, we subcutaneously injected the recombinant MHCC97-H and Hep3B cells into nude mice, measured the tumor volume each week for 4 weeks and drew the tumor growth curves. As shown in Figure 3A, miR-146b-5p over-expression significantly repressed xenograft tumor growth, whereas miR-146b-5p knockdown promoted tumor growth (*P*<0.01, respectively, Figure 3A). Determined by Ki-67 IHC staining and TUNEL assays, miR-146b-5p was demonstrated to inhibit proliferation (*P*<0.01, respectively, Figure 3B) and induce apoptosis (*P*<0.01, respectively, Figure 3C). Moreover, we established the lung metastasis mice model through the tail vein injection. Lung tissue sections were stained with hematoxylin and eosin. Compared with control groups, miR-146b-5p over-expression decreased the lung metastasis but miR-146b-5p knockdown increased the lung metastasis (*P*<0.01, respectively, Figure 3D).

### TRAF6 is a direct target of miR-146b-5p in HCC

**Figure 4:**
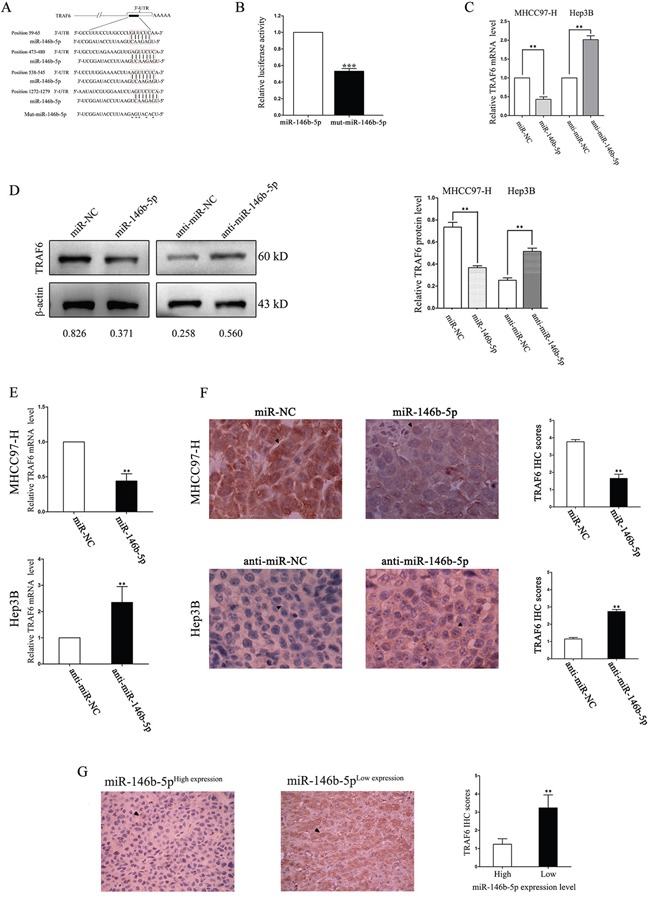
TRAF6 is a direct downstream target of miR-146b-5p in HCC **A**. The assumed miR-146b-5p binding sequences in the 3′-UTR of TRAF6. **B**. Wild-type miR-146b-5p crippled the luciferase activity that carried the 3′-UTR of TRAF6 mRNA. ^**^*P<0.001. **C-F**. Alteration of miR-146b-5p levels regulated TRAF6 expression *in vitro* (C and D) and *in vivo* (E and F). The mRNA (C and E) and protein (D and F) expression levels of TRAF6 were down-regulated through miR-146b-5p over-expression and up-regulated by miR-146b-5p knockdown. The mRNA levels were determined by qRT-PCR. The protein expression was detected by immunoblotting *in vitro* and IHC *in vivo*. ^*^P<0.01. **G**. TRAF6 expression was lower in high miR-146b-5p group than that in low miR-146b-5p group. ^*^P<0.01. All experiments were performed at least in triplicate and the data in **B-G** are presented as the (mean ± SD).

As shown in Figure 4A, TNF receptor associated factor 6 (TRAF6) was selected as a potential downstream target of miR-146b-5p through five online databases (Targetscan: http://www.targetscan.org/, picTar: http://pictar.mdc-berlin.de/, RNA22: https://cm.jefferson.edu/rna22/Interactive/, PITA: https://genie.weizmann.ac.il/ and miRanda: http://www.microrna.org/). We constructed a mutation type of miR-146b-5p and used dual-luciferase reporter gene assays to demonstrate that the luciferase activity of TRAF6 containing 3′-UTRs was inhibited only by the wild-type miR-146b-5p but not the mutant (mut) miR-146b-5p (*P*<0.001, Figure 4B). *In vitro*, we revealed that up-regulation of miR-146b-5p inhibited TRAF6 mRNA and protein expression in MHCC97-H cells, whereas down-regulation of miR-146b-5p induced TRAF6 expression in Hep3B cells (*P*<0.01, respectively, Figure 4C and 4D). We also detected the mRNA and protein expression levels in nude mice subcutaneous tumor by qRT-PCR and IHC, respectively. As shown in Figure 4E and 4F, miR-146b-5p significantly inhibited TRAF6 expression *in vivo* (P<0.01, respectively). Finally, we detected the expression of TRAF6 in HCC tissues by IHC staining. As shown in Figure 4G, the expression level of TRAF6 in the low miR-146b-5p group was higher than that in the high miR-146b-5p group (*P*<0.01, respectively, Figure 4G).

### miR-146b-5p attenuates TRAF6/p-Akt signal pathway in HCC

**Figure 5:**
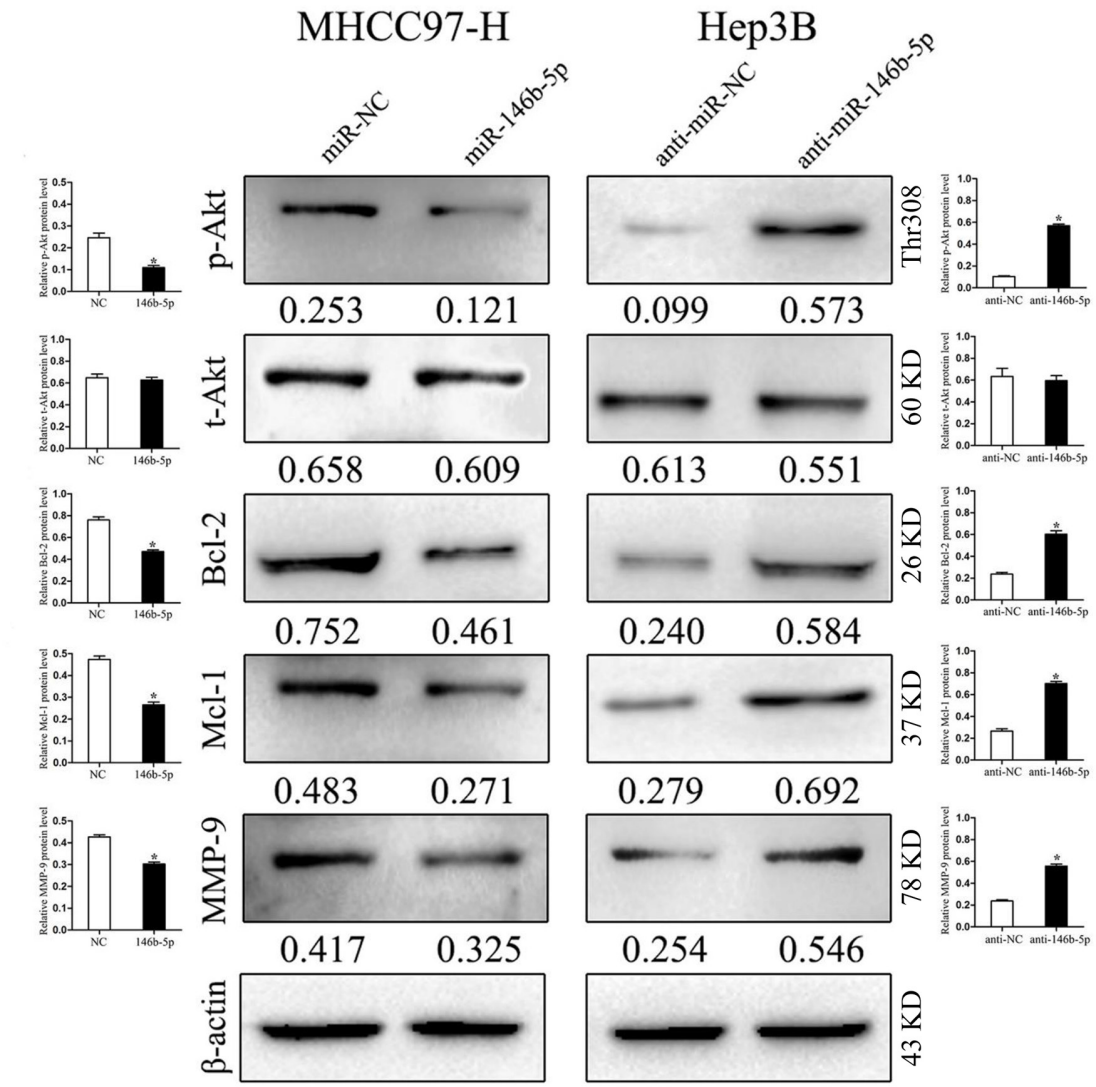
miR-146b-5p exerts its functions through inhibiting the TRAF6/p-Akt signaling pathway In MHCC97-H cells with miR-146b-5p over-expression, the phosphorylation of Akt (p-Akt) was impaired and the expression levels of Bcl-2, Mcl-1 and MMP-9 were decreased. In Hep3B cells with miR-146b-5p knockdown, enhanced the phosphorylated Akt induced the expression of Bcl-2, Mcl-1 and MMP-9. Neither over-expression nor knockdown of miR-146b-5p could find any significant changes of total Akt (t-Akt) expression. *P<0.05. Immunoblotting was performed at least in triplicate and the data are presented as the (mean ± SD).

TRAF6 is thought to achieve its functions partly through contributing the phosphorylation of Akt [17]. In order to explore whether miR-146b-5p performs its anti-cancer role though inhibiting the TRAF6-mediated phosphorylation of Akt, we detected the expression of total Akt (t-Akt) and phosphorylated Akt (p-Akt) in transfected cells. As shown in Figure 5, miR-146b-5p over-expression decreased the expression of p-Akt in MHCC97-H cells, whereas miR-146b-5p knockdown increased the expression of p-Akt in Hep3B cells (*P*<0.05, respectively, 1^st^ row). However, no significant expression changes of total Akt (t-Akt) were detected in these two cell lines (*P*>0.05, respectively, 2^nd^ row). Subsequently, we detected a series of downstream genes of the TRAF6/p-Akt signaling pathway, such as Bcl-2, Mcl-1 and MMP-9. Western blot results showed that the proteinlevels of these three molecules were down-regulated in MHCC97-H cells and up-regulated in Hep3B cells (*P*<0.05, respectively, 3^rd^ to 5^th^ rows).

### TRAF6 alters the effects of miR-146b-5p on HCC cells

**Figure 6:**
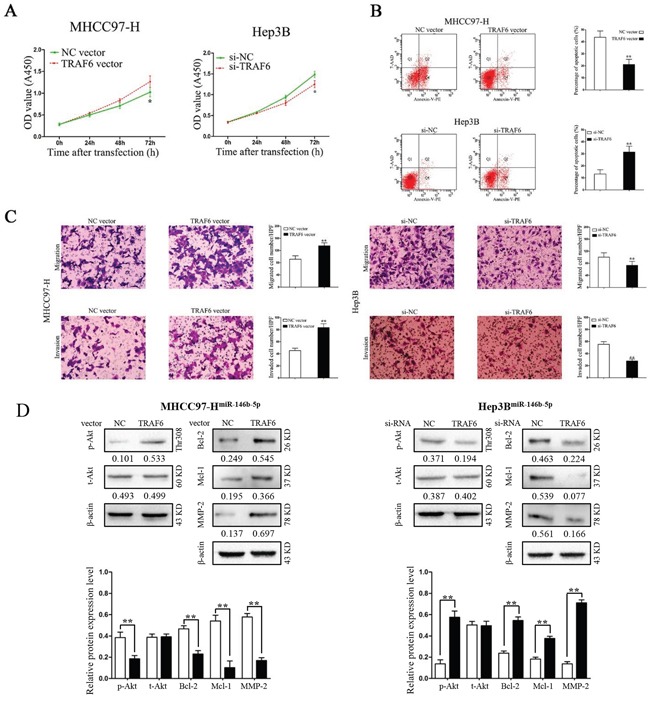
Modification of TRAF6 expression partly abrogated the functions of miR-146b-5p on HCC cells **A-C**. Modification of TRAF6 expression partly abolished the effects of miR-146b-5p on cell viability (A), apoptosis (B), migration and invasion (C) of MHHC97-H and Hep3B cells. *P<0.05, ^*^P<0.01. **D**. TRAF6 abrogated the effects of miR-146b-5p for Akt phosphorylation as well as the protein levels of Bcl-2, Mcl-1 and MMP-9. ^*^P<0.01. All experiments were performed at least in triplicate and the data in **A-D** are presented as the (mean ± SD).

In contrast, we transfected TRAF6 vectors into miR-146b-5p overexpressed MHCC97-H (MHCC97-H^miR-146b-5p^) and TRAF6 siRNAs into miR-146b-5p knockdown Hep3B cells(Hep3B^anti-miR-146b-5p^). As shown in Figure 6A, 6B and 6C, TRAF6 over-expression in MHCC97-H^miR-146b-5p^ cells promoted cell viability, migration, invasion and suppressed apoptosis; however, TRAF6 knockdown in Hep3B^anti-miR-146b-5p^ cells inhibited cell viability, migration, invasion and induced apoptosis (*P*<0.05 and *P*<0.01, respectively). Furthermore, TRAF6 abrogated the effects of miR-146b-5p for Akt phosphorylation as well as protein expression of Bcl-2, Mcl-1 and MMP-9 (*P*<0.01, respectively, Figure 6D). However, there were no significant changes for total Akt in these two cell types (*P*>0.05, respectively, Figure 6D).

### Long non-coding RNA MALAT1 serves as an endogenous sponge of miR-146b-5p in HCC

**Figure 7:**
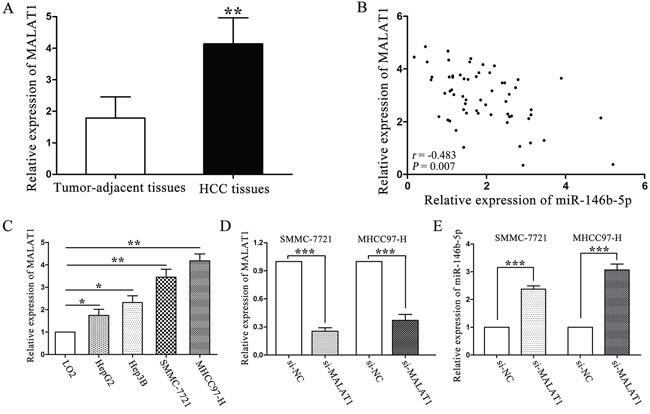
LncRNA MALAT1 negatively regulated miR-146b-5p expression in HCC **A**. Expression of MALAT1 in HCC and tumor-adjacent tissues. ^*^P<0.01. **B**. A negative correlation was verified between MALAT1 and miR-146b-5p in a cohort with 60 HCC patients (*r*=−0.483, *P=*0.007). **C**. The expression of MALAT1 in HCC cells was higher than that in LO2 cells. *P<0.05, ^*^P<0.01. **D**. Down-regulation of MALAT1 by siRNA in SMMC-7721 and MHCC97-H cells. ^**^*P<0.001. **E**. MALAT1 knockdown increased miR-146b-5p expression in SMMC-7721 and MHCC97-H cells. All experiments were performed at least in triplicate and the data in **A**, **C**, **D** and **E** are presented as the (mean ± SD).

Studies have shown that long noncoding RNAs (lncRNAs) act as an endogenous sponge of microRNAs. We used Starbase v.2.0 to predict that a long non-coding RNA, metastasis-associated lung adenocarcinoma transcript 1 (MALAT1), had a complementary sequence of miR-146b-5p. We found that the expression of MALAT1 was higher in HCC tissues than that in tumor-adjacent tissues (*P<*0.01, Figure 7A). Furthermore, a negative relationship was identified between these two non-coding RNAs, the correlation index was −0.483 (*P=*0.007, Figure 7B). MHCC97-H and SMMC-7721 cells which had high original MALAT1 expression levels (*P<*0.01, respectively, Figure 7C) were selected to silence MALAT1 expression by siRNA (*P*<0.001, respectively, Figure 7D). Real-time PCR results showed that MALAT1 knockdown rescued miR-146b-5p expression in MHCC97-H and SMMC-7721 cells (*P*<0.001, respectively, Figure 7E). These findings might explain why miR-146b-5p expression was lower in HCC.

## DISCUSSION

miR-146b-5p showed paradoxical roles in human cancers. It was reduced in gallbladder cancer tissues and correlated with large tumor size and inferior cell differentiation [18]. A prognostic value of miR-146b-5p was also embodied in ER-negative breast cancer patients [19], large B-cell lymphoma patients [20] and gastric cancer [21]. Interestingly, Yoon et.al reported that high expression of miR-146b-5p in the proximal resection margin tissues of gastric cancer was a strong risk factor for recurrence and poor survival [22]. In the present study, for the first time, we discovered that the expression of miR-146b-5p was significantly decreased in HCC tissues. Down-regulation of miR-146b-5p was correlated with malignant clinical features and poor survival in HCC patients. All above results indicate that decreased expression of miR-146b-5p is an unfavorable factor and may lead to the disorder of cell growth and invasion in HCC.

To confirm this hypothesis, a series of *in vitro* and *in vivo* assays were designed to investigate the biological functions of miR-146b-5p. We revealed that up-regulation of miR-146b-5p reduced proliferation, migration and invasion, promoted apoptosis and caspase 3/7 activity *in vitro*, and arrested tumor growth and lung metastasis *in vivo*. Even though miR-146b-5p promoted the proliferation and invasion of osteosarcoma cells [23], our results were still consistent with the previous reports in prostate and pancreatic cancers [24].

Functionally, TRAF6 exhibits an E3 ubiquitin ligase activity. Unlike other E3 ubiquitin ligases catalyze the synthesis of polyubiquitin chains linked through lysine-48 (K48), the chains catalyzed by TRAF6 was linked through the point of lysine-63 (K63) [25]. These unique chains can lead to the activation of TAK1 [26], MAPK [27] and many other kinases’ activity. We used dual-luciferase reporter system to confirm that only wild-type miR-146b-5p changed the luciferase activity of 3′-UTR of TRAF6 mRNA. miR-146b-5p could decrease the TRAF6 expression *in vitro* and *in vivo*. Finally, we provided a negative correlation between TRAF6 and miR-146b-5p expression in a cohort of HCC patients. Our results also showed that miR-146b-5p only imapired the phosphorylation of Akt through inhibiting the expression of TRAF6 but not influenced the total Akt expression. Because the K48 linked ubiquitination usually caused degradation of target proteins, we could partly confirm that K63 might be the catalytic site of TRAF6 for Akt ubiquitination. We also detected three downstream genes of Akt signaling pathway which could enhance cell growth and metastasis. First, the expression levels of Bcl-2 and Mcl-1 were down-regulated through inhibiting TRAF6/p-Akt activation by miR-146b-5p. Mcl-1 served as a substrate of caspase-3 [28], its down-regulation could be explained by the enhancement of caspase 3/7 activity after the miR-146b-5p over-expression. Next, our study also found that miR-146b-5p inhibited the expression of MMP-9, and that might explain why miR-146b-5p could inhibit cell invasion and lung metastasis. Furthermore, we rescued the expression of TRAF6 in MHCC97-H^miR-146b-5p^ cells and silenced TRAF6 expression in Hep3B^anti-miR-146b-5p^ cells. Our results showed that TRAF6 partly abrogated the effects of miR-146b-5p on HCC cell viability, proliferation, apoptosis, migration, invasion through reactivated Akt signaling pathway.

Previous studies showed that deregulation of lncRNAs profoundly influenced the expression of microRNAs [29]. For example, lncRNA HOTAIR contained a conserved binding site for miR-326. Silencing HOTAIR by siRNA could rescue the expression of miR-326 in human glioma cells [30]. Inspired by these studies, we identified a negative correlation between lncRNA MALAT1 and miR-146b-5p expression in HCC tissues. Down-regulation of lncRNA MALAT1 expression significantly increased the expression of miR-146b-5p in HCC cells.

**Figure 8:**
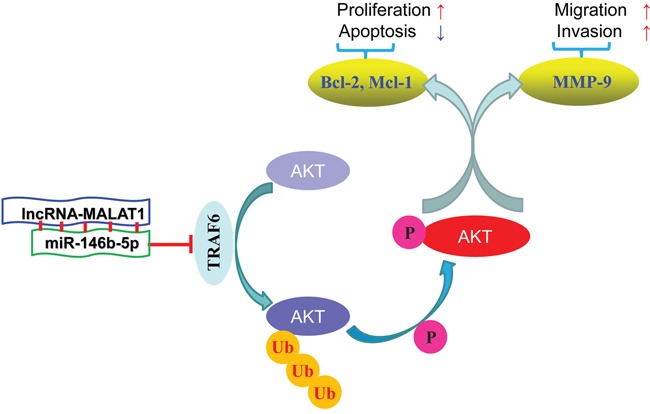
Diagrammatic sketch of the signaling pathway that miR-146b-5p arrests tumor growth and metastasis on HCC cells

In conclusion, this study demonstrated that down-regulation of miR-146b-5p in HCC tissues was related to malignant clinical features and poor prognosis. Using *in vitro* and *in vivo* studies, miR-146b-5p was demonstrated as a novel inhibitor for tumor growth and metastasis in HCC. The multiple anti-cancer functions of miR-146b-5p were due to the inhibition of the TRAF6/p-Akt pathway (Figure 8). Together, our findings suggested that miR-146b-5p could become a novel prognostic bio-marker and potential therapeutic target in HCC.

## MATERIALS AND METHODS

### Clinical specimens

A total of 60 resected HCC tissues and matched tumor-adjacent tissues were provided by the Department of Hepatobiliary Surgery, First Affiliated Hospital of Xi’an Jiaotong University (Xi’an, China) between January 2009 and May 2010. All patients received follow-up after surgery until May 2015. The median follow-up time was 31 months. The demographic, clinical and pathological characteristics were obtained from the medical records and shown in Table 1. Informed consent was obtained in accordance with a protocol approved by the Ethics Committee of Xi'an Jiaotong University.

### Cell transfection

The human immortal liver cell line LO2 and four HCC cell lines (MHCC97-H, SMMC-7721, Hep3B and HepG2) were obtained from the Institute of Biochemistry and Cell Biology, Chinese Academy of Sciences (Shanghai, China) and cultured with appropriate conditions. miR-146b-5p expression vector (HmiR0164-MR04), miRNA control vector (CmiR0001-MR04, miR-NC), miR-146b-5p inhibitor (HmiR-AN0197-AM01, anti-miR-146b), miRNA control inhibitor (CmiR-AN0001-AM01, anti-miR-NC) were purchased from GeneCopoeia Co. Ltd. (Guangzhou, China). TRAF6 cDNA clone vector (SC109844) was provided by OriGene Co. Ltd. (Beijing, China). TRAF6 siRNA (s14389), MALAT1 siRNA (4455877) and negative control siRNA (AM4611) were purchased from Applied Biosystems (Foster City, CA, USA). Transfection was performed in a six-well plate when cell confluent attained 70-90% according to the Lipofectamine^®^ 2000 (11668019, Invitrogen, Carlsbad, CA, USA) transfection manual.

### Quantitative real-time reverse transcription polymerase chain reaction (qRT-PCR)

Total RNA was isolated by TRIZOL^®^ reagent (Invitrogen, Carlsbad, CA, USA). miR-146b-5p Bulge-Loop™ miRNA qRT-PCR Primer Set (miRQ0002809-1-1) and U6 Bulge-Loop™ miRNA qRT-PCR Control Primer Set (MQP-0201) were purchased from RiboBio Co. Ltd. (Guangzhou, China). The primers of TRAF6 (F: 5′-CTATTCACCAGTTAGAGGG-3′; R: 5′-GCTCACTTACATACATACT-3′), MALAT1 (F: 5′-A TGCGAGTTGTTCTCCGTCT-3′; R: 5′-TATCTGCGG TTTCCTCAAGC-3′) and β-actin (F: 5′-AGAAAATCTGG CACCACACC-3′; R: 5′-AGAGGCGTACAGGGATAG CA-3′) were synthesized by Sangon Biotech Co. Ltd. (Shanghai, China). One-Step SYBR^®^ PrimeScript^™^ RT-PCR Kit (Perfect Real Time, RR066A, TaKaRa, Dalian, China) was used to amplify target genes. 2^−ΔΔCT^ method was applied to calculate the relative expression levels. The internal control genes were β-actin for TRAF6, MALAT1 and U6 snRNA for miR-146b-5p.

### *In vitro* cell viability, proliferation and apoptosis assays

Cancer cells were seeded into a 96-well plate with 2, 000/well in quintuplicate. Cell viability was determined by Cell Counting Kit-8 (CCK-8, E606335, Sangon, Shanghai, China) at 0, 24, 48 and 72 hours. Cell proliferation was measured by the plate clone formation assays, and the protocol we described previously [31]. Apoptotic cells were stained at the time of 72 hours’ transfection with Annexin V-PE/7-AAD apoptosis detection kit (KGA-1017, KeyGEN, Nangjing, China). 2×10^5^ cells were suspended with 50 μL binding buffer, and received the following treatments: 5 μL/sample 7-AAD with 15 minutes incubation; 1 μL/sample Annexin V-PE and 450 μL/sample binding buffer with 15 minutes incubation. Positive stained cells were detected by BD FACS Canto II Flow Cytometer (Becton Dickinson, Franklin Lakes, NJ, USA). The caspase-3/7 activity assay was measured using an Apo-ONE^®^ Homogeneous Caspase-3/7 Assay (Promega, Madison, WI, USA) in accordance with our previous description [32].

### Transwell chamber models

The 8 μm pore-sized transwell inserts (Nalge Nunc, Penfield, NY, USA) were used to detect cell migration, or coated with matrigel (BD Biosciences, Franklin Lakes, NJ, USA) at 1 mg/mL on the inner layer to detect cell invasion. Cells were re-suspended with reduced serum DMEM medium (Gibco, Carlsbad, CA, USA) and added into upper-chamber, and a 750 μL completed DMEM medium was added into the lower-chamber, then incubating for 24 hours. Cells were fixed in 4% paraformaldehyde for 2 min and stained with 0.3% crystal violet. Migrated or invaded cells on the under-surface were counted under a light microscope.

### *In vivo* tumor growth and metastasis assays

4 weeks BALB/c nude mice were purchased from the center of laboratory animals of Xi’an Jiaotong University. 5×10^6^ cells were suspended in 100 μL PBS and injected into the subcutis. Width and length of the neoplasm were measured with calipers every week. Tumor volume was calculated with the formula: π×length×width2/6. 4 weeks later, mice were excused. 100 mg tissue was collected from every sample to isolate total RNA. Neoplasms were fixed with 4% paraformaldehyde, embedded in paraffin and made into 4 μm slices. Ki-67 antibodies (#9027, CST, Danvers, MA, USA) were used to determine cell proliferation by immunohistochemical (IHC) staining as we described previously [33]. *In situ* cell death detection kit, POD (Roche, Mannheim, Germany) was used to detected cell apoptosis according to the manufacturer's instruction. The scores of TUNEL positive staining cells were expressed as the following grades: 0, <5%; 1, 6%–25%; 2, 26%–50%; 3, 51%–75%; and 4, >75%.

1×10^5^ cells with 100 μL PBS were injected into mouse systemic circulation through the tail vein. Lungs were obtained after four weeks feeding and fixed with 4% paraformaldehyde. 4 μm consecutive sections were made and stained with hematoxylin and eosin. The number of metastatic clusters was calculated to analyze the effects of different treatments.

### Luciferase reporter assay

The 3′-UTR region of TRAF6 was inserted into pmiR-RB-Report^™^ vector (RiboBio, Guangzhou, China). miR-146b-5p and recombinant vectors were co-transfected into MHCC97-H cells with Lipofectamine^®^ 2000. Ranilla luciferase activity was detected using the dual-luciferase reporter assay system (Promega, Madison, WI, USA) to reflect the binding between miR-146b-5p and the 3′-UTR region of TRAF6. Firefly luciferase activity was served as the internal control.

### Immunoblotting

Cells were dissolved by RIPA reagent (HEART Biotech, Xi’an, China). Protein levels were quantified by BCA protein assay kit II (#5000002, BIO-RAD, Hercules, CA, USA). Lysates (40 μg protein) were separated by 10% SDS-PAGE gel and transferred onto a PVDF membrane (IPVH00010, Millipore, Billerica, MA, USA). Primary antibodies of TRAF6, Akt (#4691), Phospho-Akt^Thr308^ (#13038) and β-actin (#3700) were purchased from CST (Danvers, MA, USA). Primary antibodies of Bcl-2 (12789-1-AP), Mcl-1 (16225-1-AP) and MMP-9 (10375-2-AP) were purchased from PROTEINTECH (Rosemont, IL, USA). PVDF membranes with certain proteins were incubated with matched antibodies (diluted as 1:1000) overnight. Secondary horseradish peroxidase-conjugated anti-rabbit or anti-mouse antibodies (diluted as 1:5000, ABGENT, San Diego, CA, USA) were used to bind matched primary antibodies. Protein expression levels were visualized by Enhanced chemiluminescence reagent (WBKLS0500, Millipore, Billerica, MA, USA) and calculated by Image J software.

### Statistical analysis

Measurement data is presented as Mean ± SD and processed by the SPSS statistical package for Window Version 21 (SPSS Inc., Chicago, IL, USA) and GraphPad Prism 5 software (GraphPad Inc., San Diego, CA, USA). Student's t test or one-way ANOVA were used to analyze the difference among/between sample groups. Patients’ survival was reflected by Kaplan-Meier and analyzed by log-rank test. Person correlation analysis was used to evaluate the relationship between miR-146b-5p and MALAT1. *P*<0.05 was considered to be statistically significant.
